# Clustering analysis of Yue opera character tone trends based on quantum particle swarm optimization for fuzzy C-means

**DOI:** 10.1371/journal.pone.0313065

**Published:** 2025-01-24

**Authors:** Yuhang Zhang, Xiaofeng Wu, Jiawei Xu, Zihao Ning, Xiao Han

**Affiliations:** 1 School of Social and Behavioral Science, Nangjing University, Nangjing, RP China; 2 College of Music, Nanjing Normal University, Nangjing, RP China; 3 College of Music, Communication University of China Nanjing, Nanjing, RP China; 4 College of Automation Engineering, Nanjing University of Aeronautics and Astronautics, Nanjing, RP China; University of Aizu, JAPAN

## Abstract

This study develops an innovative method for analyzing and clustering tonal trends in Chinese Yue Opera to identify different vocal styles accurately. Linear interpolation is applied to process the time series data of vocal melodies, addressing inconsistent feature dimensions. The second-order difference method extracts tonal trend features. We introduce a fuzzy C-means clustering method enhanced by quantum particle swarm optimization (QPSO) to manage data uncertainties, improving classification accuracy and convergence speed. Additionally, we employ a cross-correlation function to eliminate uncertainties from tonal transition redundancies. We designed a detection algorithm using trend data to validate our clustering method, thereby enhancing the accuracy of the analysis of tonal ranges and potential models. This method detects whether Yue Opera adheres to traditional rhythmic norms and models the regularity of musical tones and vocal patterns. Simulation results reveal that our approach achieves a 91.4% accuracy in classifying vocal styles, surpassing traditional methods and demonstrating its potential for identifying various styles. This research offers technical support for Yue Opera music education and interdisciplinary research. The findings enhance the quality of artistic creation and performance in Yue Opera, ensuring its preservation and development.

## 1. Introduction

The singing techniques in Yue opera are uniquely designed to align with the script’s textual intonation, weaving together the melody and rhythm with the script’s content [1]. Any mismatch between planned intonation and actual singing can lead to sound pattern misinterpretation, thereby affecting word recognition and diminishing the melody’s aesthetic quality. This issue complicates accurately capturing the tonal essence of Yue opera lyrics. The study aims to categorize and analyze the singing patterns associated with Yue opera’s eight tonal classes to develop precise models for each. The ultimate objective is to ensure a seamless integration of tonal arrangement and vocal execution, maintaining the lyrical rhythm traditions of Yue opera.

In the composition of Yue opera, the conflict between the requirements of musical melody and the influence of dialectal tones can result in discrepancies between the intonation of lyrics and the tune. This leads to misinterpretations and negatively impacts the aesthetic and creative aspects of Yue opera. Subjective judgments in manual analysis are vulnerable to uncertainties introduced by transitional tones and mixed tones in intonation, making it challenging to accurately describe the vocal trends of character characteristics. In this paper, ensemble mapping is considered to digitize the melody of character vocals and Tonal trend. Digitizing Yue opera tones aids cluster analysis, identifying patterns in unique Yue opera tones and assessing the clustering technique’s efficacy. However, the tonal data of mapped characters display certain traits:

(1) The tonal patterns in Yue opera exhibit variability in melodic length, introducing a degree of randomness to the feature dimensions of the data. This variability means the mapped data cannot be directly employed in cluster analysis. It is also crucial to filter out extraneous information from the character tonal data, focusing solely on the tonal trend.

(2) Since Yue opera involves changes in plate structures, where the plate structure not only dictates the rhythm but also combines with specific singing styles, variations in singing styles often coincide with plate structure transitions. This introduces uncertainty or parts considered as "noise" in the data, adding dimensions and complexity. These patterns affect the rhythm and singing style, and their changes often correspond to changes in vocal style. These "noises" characterize the complexities that notated scores cannot fully represent. The analysis of melodic trends is crucial for accurate and effective data analysis.

Due to the aforementioned challenges, the complexity of clustering Yue opera vocal data is increased. Clustering algorithms are unsupervised learning methods used to group data points into multiple clusters, where data points within the same cluster are highly similar, while those in different clusters have low similarity [2]. Commonly used unsupervised learning analysis methods include K-means clustering, hierarchical clustering, and density-based clustering [3]. Yin et al. applied a density-oriented BIRCH clustering method in medical image segmentation, overcoming the influence of complex backgrounds on medical image segmentation and improving the speed and accuracy of segmentation results [4]. Briggs et al. proposed a federated learning approach that introduces hierarchical clustering steps to enhance training on non-IID (non-independent and identically distributed) data [5]. Zheng et al. developed an innovative subspace clustering technique that leverages non-negative and low-rank representation, specifically for identifying cell types in single-cell RNA sequencing data. This method has demonstrated enhanced robustness and accuracy across various datasets [6]. Considering the randomness of feature dimensions and uncertainties at the end of vocal data in Yue opera, ordinary clustering methods with randomly generated initial cluster centers can significantly reduce the accuracy, convergence, and speed of the algorithm.

Pal et al. introduced a novel energy-efficient clustering technique based on a genetic algorithm with a newly defined objective function. Simulation results demonstrate the method’s superior effectiveness in enhancing wireless sensor network performance compared to state-of-the-art methods, namely SEP, IHCR, and ERP [7]. Xiong et al. presented an optimization method based on dynamic partial clustering for recovering spectral reflectance from camera response values. The approach adaptively applies Euclidean distance weighting and polynomial expansion models in the clustered subspace to improve spectral recovery accuracy [8]. Haeusser et al. proposed a novel end-to-end clustering training scheme for a neural network without labels by associating centroid variables with the input image through a cost function that jointly optimizes these variables and network parameters [9].

This paper introduces the fuzzy C-means (FCM) clustering algorithm, which assigns probabilities or membership degrees to each data point, avoiding rigid assignment to a specific category and addressing uncertainty in the tonal trend data of Yue opera vocalizations. To handle natural grouping and segmentation challenges in virtual data, U Qamar et al. employed the FCM algorithm to segment images of Charon, a moon of Pluto, measuring the information content of various geological features [10]. J Shankar et al. proposed a FCM clustering algorithm combined with the Bat optimization algorithm (BOA) for segmenting magnetic resonance imaging (MRI) images [11]. This study adopts the quantum particle swarm optimization (QPSO) algorithm, where data points are depicted in quantum states, aiming to boost the convergence rate toward near-optimal solutions. This approach endows the QPSO with strengths, including robustness, high precision, and superior global search abilities, particularly in optimizing clustering tasks.

The algorithms mentioned are utilized to cluster the eight features of Yue opera vocalizations. Nevertheless, uncertain interference elements at the end of the sample data features diminish the classification accuracy. Chao Pan tackled the issue of improving specific speech signals in challenging acoustic settings using microphone array beamforming technology. The problem is formulated as a convex optimization problem, and its solution yields an interference control maximum noise reduction (ICMR) beamformer under the specified interference attenuation level. Adjusting the interference attenuation factor enables the ICMR beamformer to achieve positive interference reduction or even complete elimination, but this adjustment may result in reduced additional noise suppression or even noise amplification [12]. Zhiwei Wang proposed the Siamese Octave convolution module to enhance the noise resistance of CNN features and further improve the self-similarity of template feature maps and search feature maps. Simultaneously, a novel cross-correlation fusion method is introduced, enhancing the multi-scale nature of cross-correlation results by aggregating local and global contextual information, thereby providing better adaptability under various noise levels. This method can be applied to eliminate uncertain interference factors introduced by the end-of-sound transitions in Yue opera vocalizations, separating redundant transitions, and accurately identifying the melodic patterns of vocalizations [13].

This paper is structured as follows: Section two introduces Yue opera character tones data, providing a mathematical analysis of the challenges encountered. Section three explores the optimization of character tone data features and the extraction of tonal trend features, introducing a detection approach that utilizes QPSO for fuzzy C-means and cross-correlation function analysis. Section four conducts comparative experiments using real-world data to assess the detection algorithm’s efficacy.

## 2. Problem description

### 2.1. Exploring the correlation between tone pitch and melodic structure

In Yue opera, the "character tone trend" denotes the unique melodic contours found in the opera’s lyrics, which differ based on the character tunes used [14]. This concept is influenced by the interaction between the inherent melodic shapes of the character tones and the musical tunes associated with the lyrics.

In Yue opera, the "unique" melody pertains to the pitch sequence associated with a single sung syllable, characterized by a non-sequential arrangement of pitches of different lengths. The term "yinshi" describes the melodic pitch trend or intonation. To create an intonation dataset, the pitch sequence *δ* in Yue opera Tonal Characteristic trend is represented as any element *δ* in the pitch set *Z* = {*F*,*G*,*a*,*b*,*c*,*d*,*e*,*f*,*g*,*a*^1^,*b*^1^,*c*^1^,*d*^1^,*e*^1^,*f*^1^,*g*^1^}, and a mapping A is established to obtain the numerical pitch set *X’*.

$$A:Z\to Z^{'}$$ (1)

For any element in set *Z*’ = {−8,−7,−6,−5,−4,−3,−2,−1,0,1,2,3,4,5,6,7}, the different permutations of *δ*’ reflect the tonal pitch trends of character vocals, forming the tonal trend dataset and constituting the artistic dataset. The specific mapping relationships are detailed in the **Table 1**.

**Table 1:** The mapping relationship from *Z* to *Z*’.

Set	Mapping relationship from *Z* to *Z*’															
*δ*	E	F	G	a	b	c	d	e	f	g	a^1^	b^1^	c^1^	d^1^	e^1^	f^1^
*δ*’	-8	-7	-6	-5	-4	-3	-2	-1	0	1	2	3	4	5	6	7

In **Table 2**, the mapping represents a customized numerical encoding system designed to map musical notes to numbers. This encoding approach might have been designed for specific data processing or analytical purposes, differing from the standard scientific pitch notation and tailored for the specific application scenarios used in the research. Pitch is named according to the Scientific Pitch Notation, and numerical encoding is done based on corresponding digital musical symbols. In Yue opera, some pitches do not conform to the standard semitone intervals typical of non-Western musical systems. These are considered pitches that exceed the numerical music adjustment range. For pitches beyond the range of numerical musical alterations, they are calculated in ascending or descending order of pitch from low to high, with adjacent numbers increasing or decreasing by 1. In digital music, ’0’ represents a rest, indicating no pitch information, and is therefore not included in pitch notation.

**Table 2:** The mapping relationship from *θ* to *θ*’.

Set	Mapping relationship from *θ* to *θ*’							
*S*	*α* ^0^	*α* ^1^	*α* ^2^	*α* ^3^	*β* ^0^	*β* ^1^	*β* ^2^	*β* ^3^
*S*’	1	2	3	4	5	6	7	8

The "Yue" tonal pattern *θ* corresponds to the pitch value of a single sung word, where θ is any element in the set *S* = {*α*_1_,*α*_2_,*α*_3_,*α*_4_,*β*_1_,*β*_2_,*β*_3_,*β*_4_}. To establish a mapping relationship between tonal patterns in Yue opera and the pitch dataset in a classification algorithm, the following tonal pattern mapping is proposed:

$$B:S\to S^{'}$$ (2)

In this context, *θ* represents a mapping from the set of tonal categories to the set of numerical tonal values *S*. Using *θ* to denote any element from the set *S* = {1, 2, 3, 4, 5, 6, 7, 8}, such that *θ*’ encompasses all tonal categories within the set *S*. The mapping relationship is detailed in **Table 2**. Through the mapping of A and B, the pitch and tonal categories of Yue opera can be represented in a data table, facilitating the classification of tonal patterns in musical expressions. To uncover the patterns between the melody of Yue opera and its tonal categories, we have designed algorithm ⊗.

Q=⊗(K) (3)

where *Q* represents tonal category labels defined by the elements *ξ* in set *P*, and *K* represents the dataset of Yue opera melody, with any sample data *K*_*i*_(*i* = 1,2,…,*N*) where *N* is the total number of samples, representing an unordered arrangement of melodies constituted by elements *θ* from any section of the set *Z*’. The objective is to design a melody classification algorithm denoted by ⊗.

**Fig 1:**
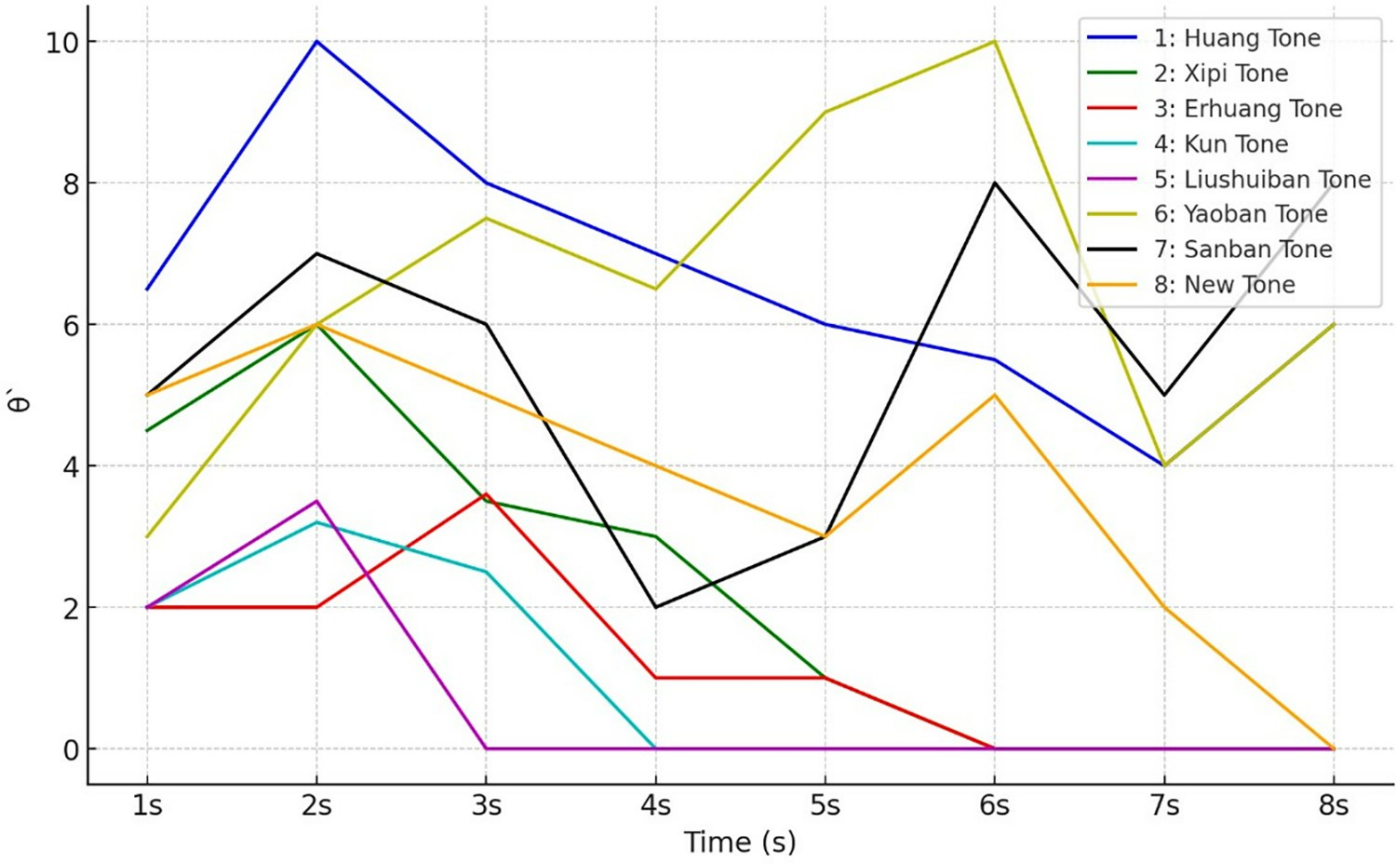
Sample data of Yue opera character tone melodies *K*_*i*_(*θ*’ = 0).

For instance, in Eq (3), a subset of the tonal category *θ*’ from label *Z* is considered, and the pitch values *θ*’ are illustrated in **Fig 1**. After the establishment of the People’s Republic of China, Yue opera developed from eight major schools to thirteen major schools. However, the eight major schools from Shaoxing are still recognized as the mainstream Yue opera schools [15].

It can be concluded from **Fig 1** that curve 1 represents the character tone melody composed of the elements [1, 2, 3, 4] in constructing sampled data in the tone trend classified recognition algorithm. Lines 2 to 8 correspond to through, signifying that the mapping set from **Table 1** and **Table 2** has effectively converted the melodic sequences into Yue opera melody data K for subsequent classification and recognition algorithms. Nonetheless, in **Fig 1**, the melodic sequences’ lengths are inconsistent, specifically {8, 5, 5, 3, 2, 8, 8, 7}, leading to non-uniform feature dimensions and introducing randomness. Furthermore, the endings of to, belonging to the same tonal category, display varying patterns. Consequently, direct clustering of the Yue opera melody dataset becomes impractical.

### 2.2. Interference factors at the end of the vocal cavity

In Yue opera, "cross-melody interference" refers to the discordance or misalignment in pitch or rhythm experienced during singing, often due to transitions between different singing styles (cross-melody) [16]. This phenomenon is an interfering factor that impacts the ending portions of data sequences in a melody dataset. The subsequent development of a classification algorithm for character tone melodies is outlined as follows:

Z=⊗(K(λ)) (4)

where *λ* signifies the interfering factor, and K(*λ*) denotes the dataset of Yue opera melodies with end-of-sequence interference, represented as K. Eq (4) states that the category of characteristics tones *θ*’ in the dataset K is influenced by the interfering factor resulting from pitch transitions. The symbol K represents an undisturbed Yue opera melody dataset, which includes various pitch patterns and melody samples intended to facilitate music *λ* analysis and classification. Additionally, *λ* introduced in Eq (4), signifies disturbance or noise, indicating that some music samples experience uncertain disturbances toward their endings, potentially affecting the clarity of melody classification and analysis. Due to the indeterminacy of the position and magnitude of pitch transitions in Yue opera vocal melody and the difficulty in establishing a mathematical model for pitch transitions, there are options other than direct separation. As depicted in **Fig 1**, the data *K*_1_∼*K*_8_ categorized with vocal category $\theta^{\prime }$ = 0, displays diverse pitch trends at the end, with *λ* regarded as the uncertainty-interfering factor within the dataset of characteristics tone melodies.

Consequently, it can be inferred that the concluding features of melodic data *χ* within the identical tonal category of the character tone manifest notable distinctions. The tonal category cannot be fully captured by the data features annotated based on prior experience in Eq (3), leading to unreliable dataset labels that adversely affect the subsequent training performance of classifiers. The classification algorithm ⊗ can overcome data labeling challenges by utilizing unsupervised learning, specifically Fuzzy C-means clustering, for character tone recognition tasks based on melodic patterns.

### 2.3. Inconsistent syllable tone feature dimensions

In Yue opera character tone melodies, the order of arrangement of pitch values *θ*’ and the number of pitch values forming the melody are arbitrary [17]. The dataset of character tone melodies, denoted as *χ*, displays the characteristic of inconsistent feature dimensions. Illustrated in **Fig 1**, for specific data associated with the identical character tone category *θ*’ = 0, the feature dimensions are {8, 5, 5, 3, 2, 8, 8, 7}, signifying a dataset *χ* of non-fixed length influenced by the interference factor *λ*. The variability in feature dimensions presents a challenge, as it needs to align with the requirement for uniform input data dimensions, which is crucial for the effective design of classification algorithms.

Dimensionality reduction and expansion are frequently employed to address the issue of inconsistent dimensions. Reducing a two-dimensional problem to one dimension allows for using certain features in classification. Conversely, dimension expansion enhances the separability of data categories due to increased feature dimensions. However, the inter-feature correlations within each dimension could result in an overly complex feature set for classification. Consequently, a strategic approach is required to extract pitch trends, ensuring the retention of essential feature information while normalizing data dimensions for consistent analysis.

## 3. Method

This section delves into the development of an algorithm aimed at detecting pitch trends and characteristics-based tonalities in Yue opera singing. With the characteristics-based melodic dataset *χ* is input, the algorithm denoted as ⊗, is specifically crafted to extract the corresponding pitch trends of a melody. This facilitates the recognition and detection of tonalities in Yue opera singing trends.

Linear interpolation and second-order difference methods extract pitch trend features with standardized dimensions. Given the uncertain interference factors in Yue opera singing trend data, selecting initial cluster centers becomes challenging. The QPSO algorithm is introduced to tackle this challenge. Applying this approach to optimize initial centers in the FCM algorithm enhances the global search for FCM’s optimum, thereby improving clustering. The cross-correlation function (CCF) algorithm effectively captures pitch trends in Yue opera character characteristics. By combining feature extraction, quantum optimization, and accurate modeling, a Yue opera singing tonality detection algorithm is devised. This algorithm is capable of assessing if Yue opera performances conform to the established pitch trends specific to character characteristics.

### 3.1. Optimization and extraction of tone trend features in Yue opera character tone data

This section describes the creation of an algorithm aimed at identifying pitch trend categories within the phonetic patterns of Yue opera. When employing the phonetic melody dataset *χ* as input, the varying feature dimensions and the uncertain interference factor *λ* within the phonetic melody data *χ* can lead to diminished accuracy in classification and detection and decelerate the convergence of the algorithm. Employing dimension expansion through linear interpolation and extracting pitch features using the first-order difference method eliminates redundant information in the phonetic melody dataset *χ*. This process aims to enhance the performance of subsequent classification and detection algorithms.

#### 3.1.1 Dimension expansion through linear interpolation

**Fig 2:**
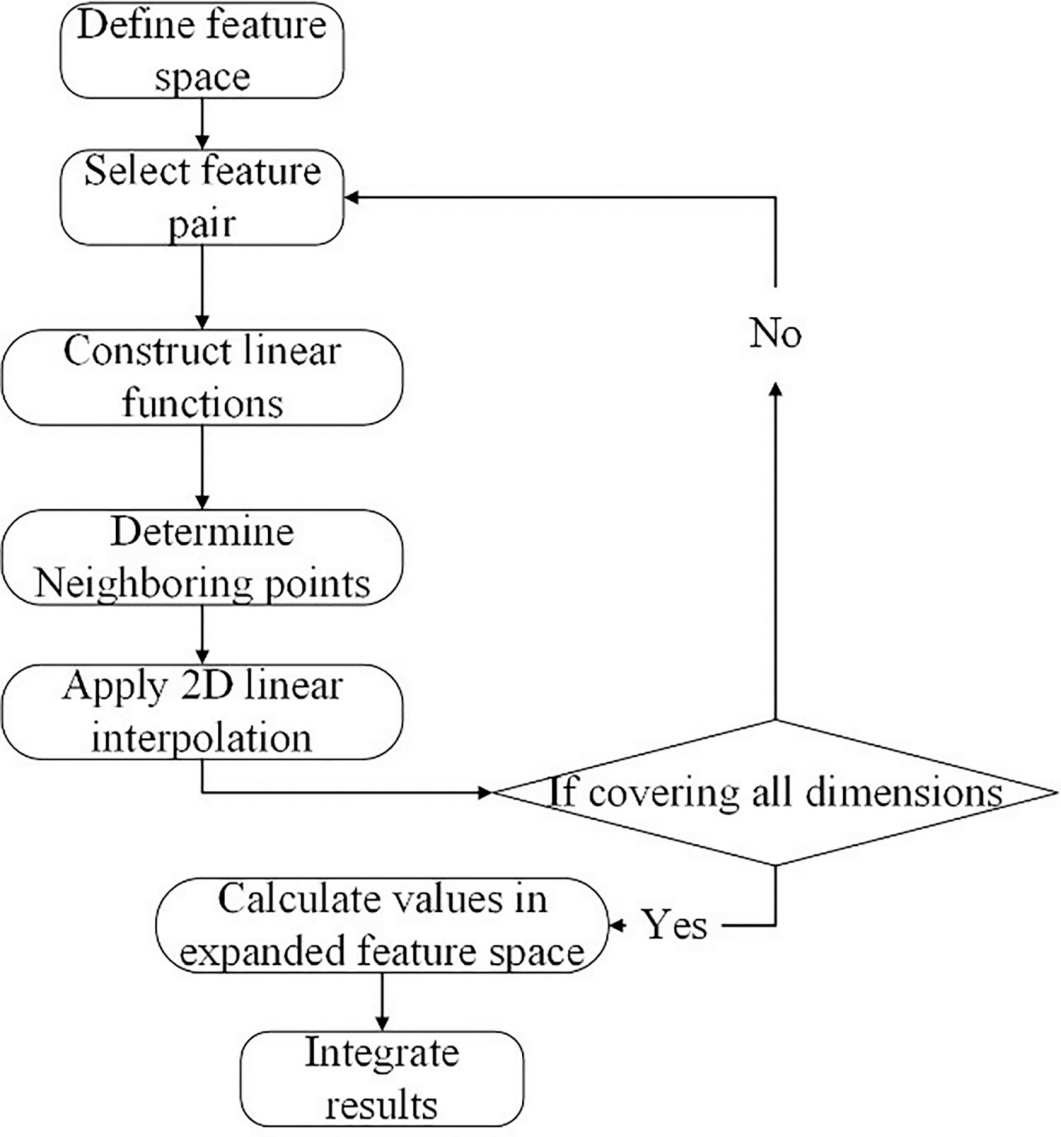
Linear interpolation dimension expansion algorithm flowchart.

We employ the linear interpolation method for dimension expansion to resolve the problem of inconsistent feature dimensions in dataset K. Linear interpolation extends the dimension of data K, which has inconsistent feature dimensions, enabling uniform normalization of all data dimensions without altering feature information. Linear interpolation dimension expansion algorithm flowchart is presented in **Fig 2**.

To perform linear interpolation on a sample data point with a feature dimension of d (*i* = 1, 2,…, *N*, and *N* is the total number of sample data), we choose two dimensions from this feature, denoted as K_*in*_ = (K_*i*1_,⋯,K_*in*_,⋯,K_*id*_)(*n*≤*d*) and K_*im*_ = (K_*i*1_,⋯,K_*in*_,⋯,K_*id*_)(*m*≤*d*). Assuming the *d* values represent a linear function within an interval of length d’ (where d’ is the desired extended dimension), we construct a linear function using the values of this function at points where *θ*’(*n*) takes values within the d’ interval. *θ*’(*n*) represents the mapped pitch data points, used to denote specific pitch values within the character tone melodies. These values are derived by applying mathematical mappings to the original pitch data, with the objective of quantifying these pitches for easier analysis and processing.

**Fig 3:**
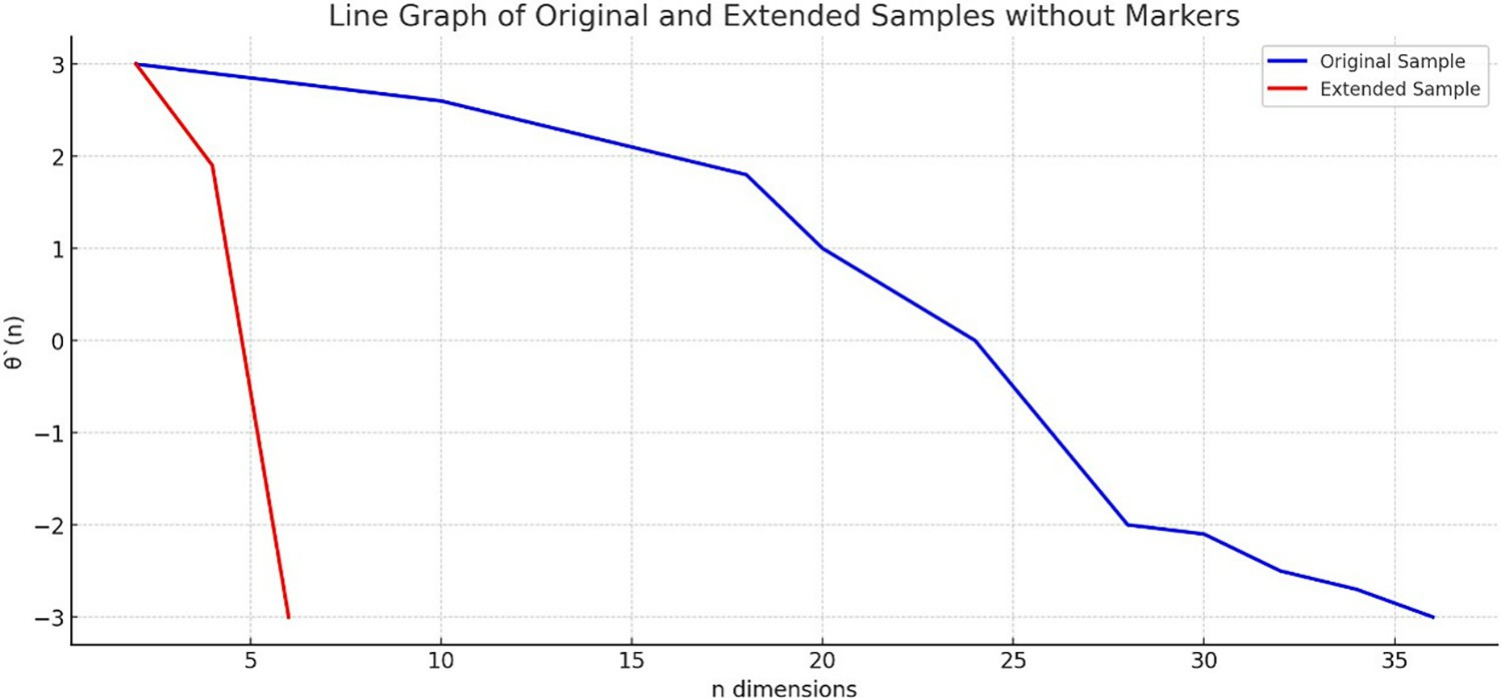
Compare data after dimensionality expansion with original sample.

The interpolation formula is as follows:

$$\theta^{'}(n)=K_{i1}+\frac{K_{i2}-K_{i1}}{n}(n-1),n\in [1,2,\cdots ,v]$$ (5)

where K_*i*_ represents the *i*_th_ sample data of dataset, K_*i*1_, K_*i*2_ represents a linear interpolation between the first and the second eigenvalue, v denotes the number of features expected to expand between the two. Through linear interpolation, the dimension of the feature vector of a certain initial feature with d = 4 in the character tone melody dataset K is expanded to d’ = 36, which is the highest dimension in the dataset, as shown in **Fig 3**. As can be seen from Fig 3, the dimension of the initial dataset is extended to 36 dimensions without changing the pitch θ^‘, and all the information of the initial dataset is retained completely.

#### 3.1.2 Feature extraction using first-order difference operation

After completing two-dimensional linear interpolation, feature extraction through first-order differencing (first-order difference operation) is a standard method, especially in signal processing and time series analysis. Although the dimension of the extended phonetic melody dataset *χ* has been standardized through linear interpolation, redundant information still exists, which can degrade the accuracy and slow down the convergence speed of subsequent classification algorithms. After dimension expansion, performing first-order differencing on the phonetic melody dataset *χ* yields the phonetic pitch. For a given dataset ot, each sample *K*_*i*_ has d features, represented as K_*i*_ = (K_*i*1_,⋯,K_*in*_,⋯,K_*id*_). The formula for first-order differencing calculates the difference between each feature K_*in*_ and its preceding feature value K_*i*(*n*−1)_. For each feature n in the sample K_*i*_,*n*∈[2,3,⋯,*d*], the first-order difference is defined as:

$$\Delta K_{in}=K_{in}-K_{i(n-1)},n\in [1,2,\cdots ,d^{'}]$$ (6)

where ΔK_*in*_ represents the second-order difference value of the nth feature of the *i*_th_ sample. Data points are represented as the first-order difference, which would be calculated as the difference between adjacent pitch points. Essentially, it means the jump size from one pitch point to the next, or the pitch change, which in musical analysis is often referred to as pitch interval.

For the entire dataset, perform a first-order differencing operation on each sample feature (except for the first feature, as it lacks a preceding feature value). This differencing operation will generate a new set of differencing feature vectors, with each vector corresponding to a sample in the original dataset:

$$\Delta K_{i}=(\Delta K_{i2},\Delta K_{i3},\cdots ,\Delta K_{id})$$ (7)

**Fig 4:**
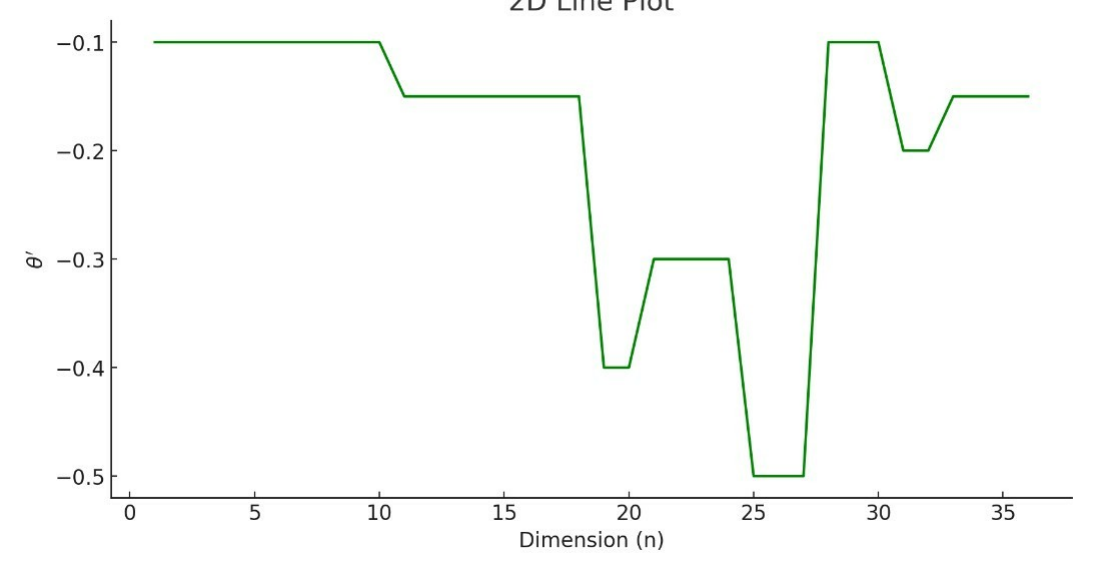
Extraction of tone trend feature *θ*’.

Since the first feature K_*i*1_ lacks a preceding value, first-order differencing typically begins from the second feature. This method is beneficial in time series analysis and signal processing as it can reveal trends and pattern changes in the data. Here, d’ represents the feature dimension after dataset expansion, and in this case, d’ is set to 36. Furthermore, given that the phonetic pitch is independent of the initial pitch, to minimize its effect on the 16 pairs of sample data, the value of the first feature in each data group is set to *θ*_*t*_(1) = 0. This refers to sixteen pairs of pitch data points selected from the voice melody dataset for experimental analysis. Each pair exemplifies a specific pitch transition, chosen to represent the dataset’s diverse changes. These pairs were critical in validating and assessing our proposed pitch trend analysis algorithm’s performance, offering insights into its accuracy and applicability in identifying and quantifying pitch variations in musical sequences. For instance, when applying first-order differencing to the phonetic melody sample data illustrated in **Fig 3**, the resultant phonetic pitch *δ* is obtained, as depicted in **Fig 4**.

The observation in **Fig 4** reveals that, utilizing the first-order differencing method on standardized 36-dimensional data, *θ*’ processes characteristics pitch melody data to extract the trend of the characteristics pitch as novel features. This process effectively eliminates redundant pitch information from the melody.

After expanding the dimension of the initial characteristics pitch melody dataset and extracting pitch trend features, the obtained characteristics pitch trend dataset is recorded as K’. It fully preserves the required pitch trend K’ feature information and obtains characteristics pitch trend data *θ*_*t*_ in the same dimension, making it easier for classification algorithms to cluster based on these features.

#### 3.1.3. First-order difference operation for feature extraction

By optimizing features and extracting pitch trend features from the characteristics pitch melody dataset K, a melody dataset K’ suitable for the clustering analysis of phonetic pitch trends has been obtained. This study chose Fuzzy C-means Clustering. Due to the presence of uncertainty interference factor B, randomly initialized cluster centers can easily lead to the entire convergent network falling into a local minimum solution, thereby deteriorating the clustering effect of pitch trends. Therefore, the Quantum Particle Swarm Optimization algorithm is introduced to search for an approximately optimal solution for cluster centers, as obtaining the *λ* exact model is not feasible. This eliminates the influence of *λ* on the selection of cluster centers for the pitch trend dataset K’.

### 3.2. Fuzzy C-means clustering

The uncertain interference factor present at the end of Yue opera vocal data features makes traditional pitch value labeling methods unreliable. As a result, the unsupervised learning method of Fuzzy C-means Clustering has been selected to address this challenge. Given the Yue dataset K’ = [K_*in*_], where *i* = 1, 2, …, I; *n* = 1, 2, …, *N*; *n* represents the number of samples; *m* represents the vocal variables in the samples. According to explicit criteria of FCM algorithm used to partition data points, this set can be divided into c fuzzy subsets using the fuzzy clustering algorithm, where $\theta^{'}=\{\theta_{1}^{'},\theta_{2}^{'},\cdots ,\theta_{c}^{'}\}$ represents the set of *c* cluster centers. Here, the number of clusters can be described by c, and c is significantly smaller than the total number of clustered samples.

The membership matrix H can be used to represent the results of fuzzy clustering. H = [*h*_*ik*_] represents the degree of membership of the i_th_ sample to the k_th_ cluster center, with values ranging from 0 to 1. Here, $\sum_{k=1}^{c}h_{ik}=1$. The objective function is given again:

$$J(K,\theta )=\sum_{i=1}^{n}\sum_{k=1}^{c}h_{ik}^{m}d_{ik}^{2}$$ (8)

where *d*_*ik*_ represents the distance between the i_th_ sample and the k_th_ cluster center *K*. Its mathematical formula is:

$$d_{ik}=\|x_{i}-v_{k}\|={\left(x_{i}-\theta_{k}\right)}^{T}A(x_{i}-\theta_{k}),i=1,2,\cdots ,n;k=1,2,\cdots ,c$$ (9)

At the same time, E represents a symmetric matrix. Under the assumed condition E = I, *d*_*ik*_ can be considered the Euclidean distance, where I is the identity matrix. The mathematical formula for *h*_*ik*_ is:

$$h_{ik}=\frac{1}{\sum_{J=1}^{C}{\left(d_{ik}/d_{in}\right)}^{\frac{2}{m-1}}},i=1,2,\cdots ,n;k=1,2,\cdots ,c$$ (10)

where *d*_*ik*_ = ‖*x*_*i*_−*y*_*j*_‖ represents the distance between the i_th_ center and the j_th_ data point, *d*_*in*_ = ‖*x*_*i*_−*y*_*n*_‖ represents the distance between the i_th_ center and the n_th_ data point, where n belongs to [1,∞) is a fuzzy parameter. FCM uses iterative gradient descent to calculate the centroids, and its update formula is as follows:

$$x_{i}=\frac{\sum_{j=1}^{N}\mu_{ij}^{m}v_{ki}}{\sum_{j=1}^{N}\mu_{ij}^{m}}$$ (11)

Each cluster center, denoted as *x*_*j*_, is identified by its position, distinguishing different cluster centers. The position of each data point, $v_{k_{i}}$, marks the elements within the dataset that are grouped based on their characteristics. The membership coefficient *h*_*ij*_, which measures the extent of association of each data point iii with a cluster center *j*, is crucial for reflecting the relationship strength. The fuzzifier parameter m influences the fuzziness of membership, balancing the distribution of data points across clusters by increasing or decreasing the degree of their collective belonging. Lastly, N represents the total number of data points involved, providing a scale of the dataset under analysis. Through the strategic manipulation of these variables, FCM facilitates the nuanced grouping of data, ensuring that each cluster reflects coherent and meaningful patterns inherent in the dataset.

The objective function minimized by FCM can be expressed as the sum of the weighted Euclidean distances based on membership:

$$\phi =\sum_{i=1}^{C}\sum_{j=1}^{N}\mu_{ij}^{m}{\left(\|x_{i}-y_{j}\|\right)}^{2}$$ (12)

where *J*(*K*,*θ*) and *m* represent the weighted average from each sample to the cluster center and the fuzzy weighting exponent. The clustering criterion of the FCM clustering algorithm is to calculate *K* and *θ* that minimize *J*(*K*,*θ*). However, the FCM clustering algorithm can be fundamentally viewed as a local search optimization, where a hill-climbing algorithm is employed to obtain the optimal solution during the optimization process. Therefore, it commonly faces issues of local optimal convergence and sensitivity to initial points.

#### 3.2.1. Quantum particle swarm optimization algorithm

Particle swarm optimization algorithm (PSO) is an evolutionary algorithm derived from the study of bird flocking behavior (Eberhart et al. "Particle swarm optimization." Proceedings of the IEEE international conference on neural networks. Vol. 4. 1995.). It begins optimization from an initial population with more robust global parallel search capabilities. In PSO, particles update their positions based on their individual best positions (*p*_*best*_) and the global best position (*g*_*best*_).This research aims to optimize cluster center positions to minimize the clustering objective function, which is defined as the sum of the weighted distances from data points to their respective cluster centers. Optimizing cluster center positions enhances the accuracy and robustness of clustering outcomes, particularly in scenarios involving data uncertainty and noise. The QPSO algorithm is used to approximate the optimal positions of these cluster centers. After each iteration, their velocities and positions are updated as follows

$$v_{ij}(t+1)=\varphi v_{ij}(t)+C_{1}r_{1}(t)(p_{ij}(t)-x_{ij}(t))+C_{2}r_{2}(t)(p_{ij}(t)-x_{ij}(t))$$ (13)

$$x_{ij}(t+1)=x_{ij}(t)+v_{ij}(t+1)$$ (14)

In Eq (13), "the i_th_" refers explicitly to the " i_th_ particle" in Particle Swarm Optimization (PSO). Each particle represents a potential solution, navigating within the search space. This term means the current velocity of the i_th_ particle in the j_th_ dimension, influenced by the inertia weight. The inertia weight regulates the particle’s velocity and capacity to sustain its current motion. *C*_1_ represents the Cognitive Acceleration Constant, governing how the particle’s movement in the search space is influenced by its individual historical best position (*p*_*best*_). A higher value indicates that the particle is more likely to adapt its search path based on previous successful experiences. *C*_2_ represents the Social Acceleration Constant, governing how the particle’s movement is influenced by the historically best position of the population (*g*_*best*_). Increasing this parameter enhances the likelihood of the particle moving towards advantageous positions discovered by other particles in the population. *r*_1_ and *r*_2_ are two sets of independently and identically distributed (i.i.d.) random numbers, each ranging from 0 to 1. *x*_*ij*_ and *v*_*ij*_ represent the position and velocity of the i_th_ particle in the j_th_ dimension, whereas *p*_*ij*_(*t*) and *p*_*gj*_(*t*) represent the pbest and gbest positions. The first term on the right-hand side of Eq (13) refers to i_th_, which represents the inertia of the i_th_ particle. In contrast, the second and third terms introduce guiding perturbations towards the attractor basin in the direction of particle movement. The update of the individual best position (*p*_*best*_) follows a greedy update scheme, considering the minimization of the cost objective, as shown in the following equation:

$$\begin{array}{l} x_{ij}(t+1)=x_{ij}(t)+v_{ij}(t+1)f(x_{i}(t+1))<f(p_{i}(t))\Rightarrow p_{i}(t+1)=x_{i}(t+1)\\ else\;if\;p_{i}(t+1)=p_{i}(t) \end{array}$$ (15)

where *f* is used within the PSO algorithm context and generally represents the objective function or fitness function. In this particular instance, f denotes the value of the objective function at the position. *x*_*ij*_ is the cost, and *p*_*i*_ is the individual best (personal best) of the particle. The global best is the element with the minimum cost within the individual best historical set of a specific particle. A major limitation of the standard PSO is that it cannot guarantee convergence to the optimal solution.

However, the major drawbacks of the PSO algorithm lie in premature convergence and weak local search capabilities [18]. To enhance the randomness and global search performance of the PSO algorithm, Sun and Xu (2004) proposed the Quantum-Behaved Particle Swarm Optimization [19] algorithm. It assumes that quantum-behaved particles move without a clear trajectory and can appear at any position in the feasible solution space.

In clustering Yue operatic vocal styles, uncertainty disturbance complicates the selection of initial cluster centers for Fuzzy C-means Clustering. Randomly generated centers can significantly degrade clustering performance.

$$mbest_{j}=\frac{1}{N}\sum_{i=1}^{N}p_{ij}$$ (16)

$$\phi_{ij}=\omega p_{ij}+(1-\omega )p_{ij}$$ (17)

$$z_{ij}=\phi_{ij}+\gamma |mbest_{j}-z_{ij}|\ln(\frac{1}{q})\forall$$ (18)

In Eqs (18) and (19), *m* represents a probability threshold that determines the direction of the particle’s position update. When a random number is greater than or equal to 0.5, the particle moves in one direction; otherwise, it moves in the opposite direction. This mechanism aids in exploring different regions of the solution space. *z*_*ij*_ represents the position of the i_th_ particle in the j_th_ dimension. The *q* is a random variable that simulates quantum potential uncertainty and fluctuation, allowing the particle to search the solution space extensively. mbest is the mean of pbest for all dimensions of the entire population, *q* is the local attractor for particle *i*. *w*, *q*, and *m* are independently and identically distributed random numbers uniformly distributed on [0,1]. *r* is the contraction-expansion coefficient, which changes with iterations, as shown below:

$$\gamma =0.9(\frac{iteration_{\max}-iteration_{current}}{iteration_{\max}})+0.1$$ (19)

#### 3.2.2 Fuzzy C-means QPSO

In this approach, each particle represents a *D*-dimensional candidate solution in one of the **C** clusters and can be formally represented as the matrix X:

$$X=\left[\begin{array}{ccc} x_{11} & \cdots & x_{1D}\\ \vdots & \ddots & \vdots \\ x_{C1} & \cdots & x_{CD} \end{array}\right]$$ (20)

**Fig 5:**
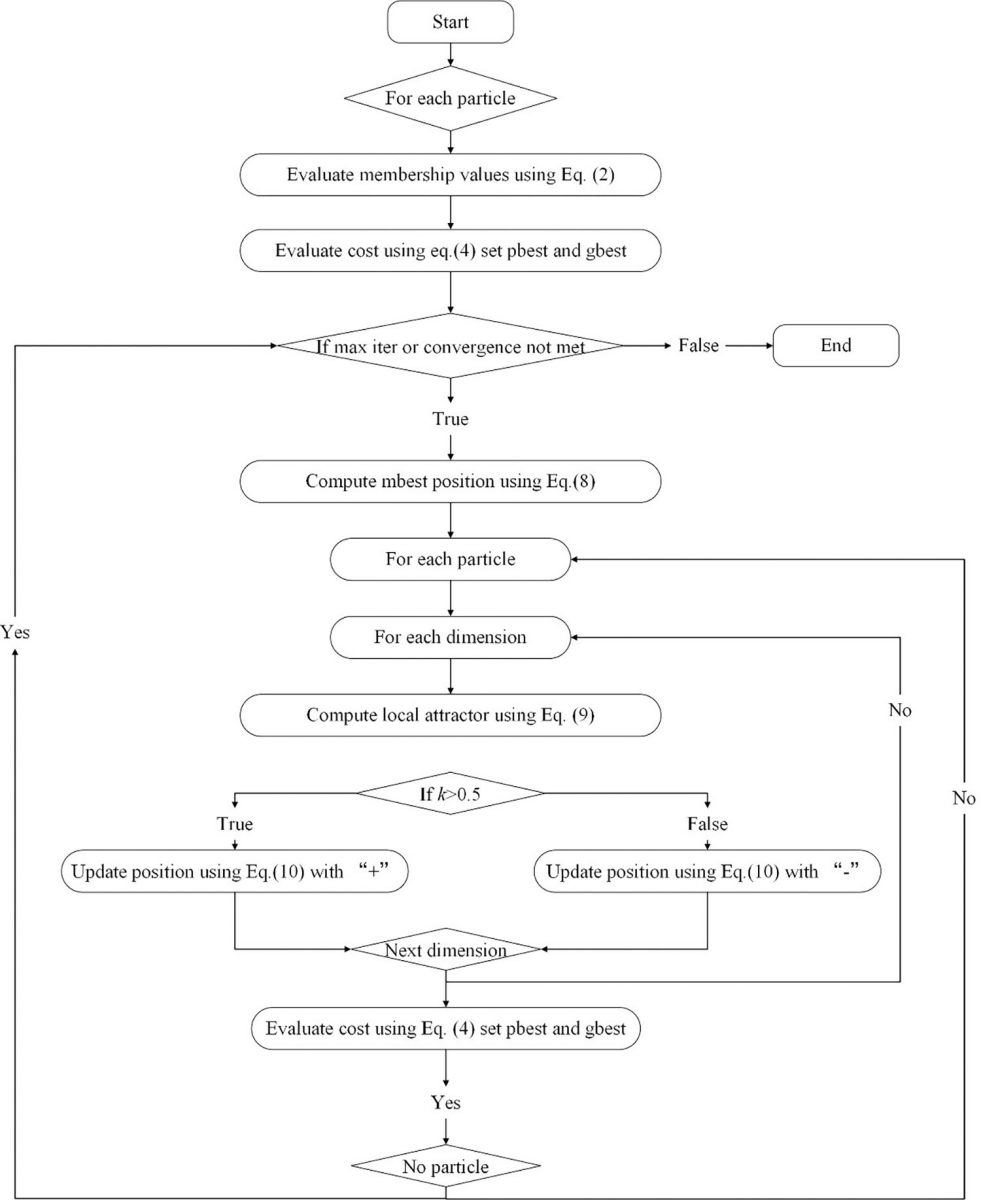
The flowchart of FCM QPSO.

**Fig 6:**
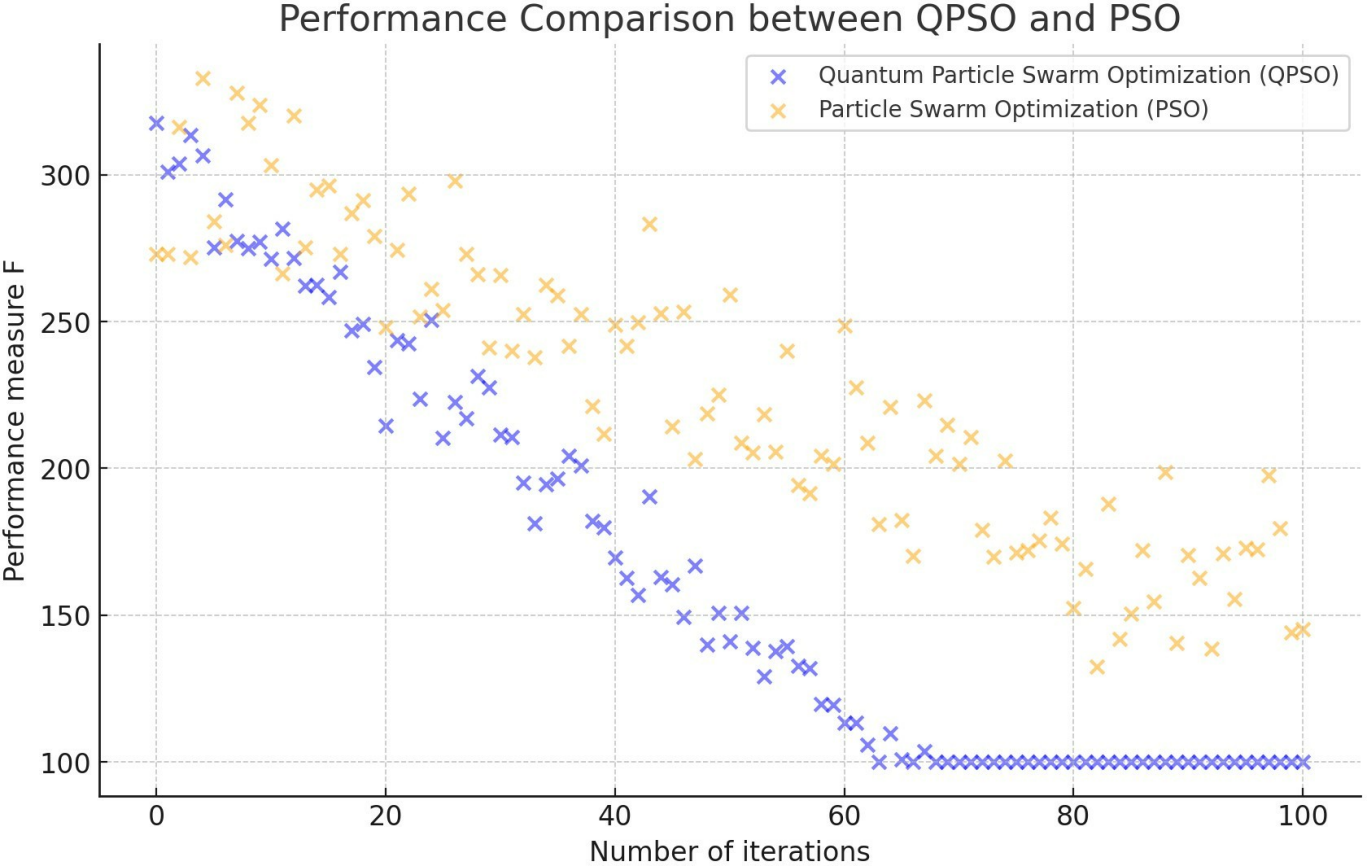
Performance comparison between QPSO and PSO.

A swarm of particles is randomly initialized, and individual best positions and the global best position are determined. Subsequently, through recursive computation using the average best positions from Eq (7) and assigning costs to each particle based on membership values and cost functions from Eqs (1) and (3), the QPSO algorithm minimizes the cost associated with each particle. It updates the candidate cluster center solutions in matrix X. The algorithm terminates if there is no improvement in the global best and the algorithm stagnates or if a predefined number of iterations is reached. By leveraging the random and non-differentiable objective function handling capabilities of QPSO within the FCM algorithm framework, it is possible to alleviate the problem of local minima stagnation in multi-dimensional search space, which may be more effective than using traditional FCM alone. The flowchart of FCM QPSO is presented in **Fig 5**:

From **Fig 6**, it can be observed that in the case of higher dimensions of the sought optimal solution, the genetic algorithm is prone to getting stuck in local optima and cannot find the global optimum approximate solution. Traditional particle swarm algorithms often converge to local optima during the later execution stages. This limits their exploration of other potentially more optimal solutions and results in redundant computations, thus slowing down the convergence rate. In contrast, these algorithms’ design of adaptive laws improves their local search capabilities. Consequently, the Quantum Particle Swarm Optimization algorithm provides a broader search range and quicker convergence speed, enabling it to achieve more accurate approximations of cluster centers compared to conventional methods. This facilitates the initial modeling of phonetic pitch *δ*.

**Fig 6** presents a performance comparison of the Quantum Particle Swarm Optimization (QPSO) algorithm with the traditional Particle Swarm Optimization (PSO) algorithm in a specific clustering task. The performance metrics in this study include convergence speed, solution quality, and algorithm stability. These metrics offer detailed insights into the efficiency and effectiveness of both algorithms in practical applications, demonstrating the QPSO algorithm’s advantages in clustering accuracy and operational speed.

Traditionally, without external disturbances, the tonal trends of Yue opera characters are categorized into eight clusters based on subjective human recognition. However, the clustering error is relatively significant when direct clustering is applied due to variations in feature values at the end of the tonal melody data for the same character tune, a discrepancy attributed to the disturbance factor λ. Based on the principles of optimizing clustering centers using the Quantum Particle Swarm Algorithm and considering the impact of disturbance factors on the tonal trends of Yue opera characters, both the traditional genetic algorithm and quantum particle swarm algorithm are employed to optimize the eight clustering centers. The particle quantity is set to 100, the inertia weight is 0.5, the learning factor is 2.0, the acceleration constant is 1.5, the convergence criterion threshold is 0.001, and the maximum iteration is set to 300. The iteration curve of the optimization algorithm is depicted in **Fig 6**. Observing Fig 6, it is apparent that when the dimension of the optimal solution for the optimization parameters is large, the outcomes of the genetic algorithm tend to get stuck in local optima, failing to identify the global optimal approximate solution. Furthermore, during the latter stages of the particle swarm algorithm, particles often converge towards local optima, neglecting to investigate other potentially superior areas, thereby leading to superfluous computations and decelerating the convergence rate.

**Fig 7:**
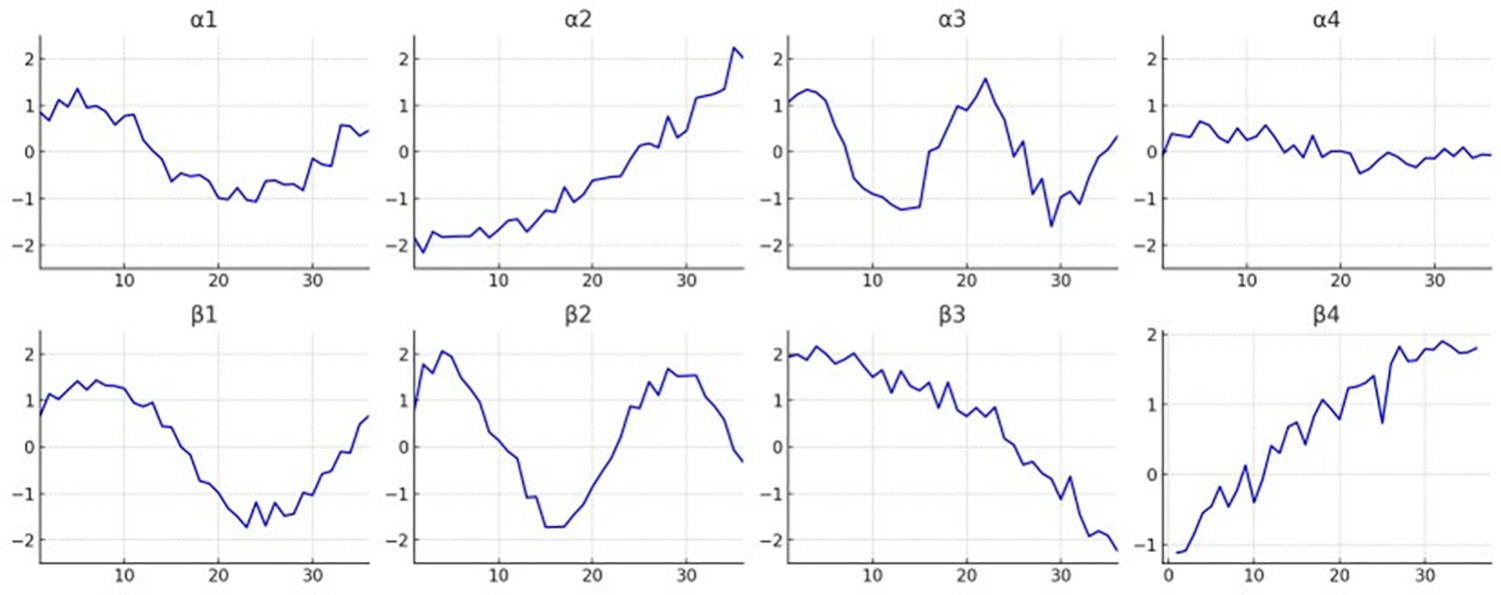
The tonal trends associated with the eight-character tunes in Yue opera.

Compared to the traditional particle swarm optimization algorithm, introducing adaptive rules in the quantum particle swarm optimization algorithm significantly enhances its capability for local search. As a result, the quantum particle swarm algorithm offers a more extensive search range and quicker convergence, yielding superior approximate results for clustering centers and facilitating the initial modeling of character tonal trends. Utilizing data features derived from the Quantum Particle Swarm Optimization algorithm, which is grounded in quantum theory, as clustering centers and setting a maximum iteration limit of 300, the Yue opera character tonal trend dataset is subjected to fuzzy C-means clustering. This process determines the clustering centers that are then displayed, with the outcomes illustrated in **Fig 7**.

Fig 7 illustrates the results of applying this clustering method, showing the classification effect of tonal trends among Yue Opera characters. The goal is to enhance the accuracy of Yue Opera vocal style recognition through precise clustering of vocal data. To reduce redundancy-induced interference in tonal transition, the algorithm employs the Cross-Correlation Function (CCF), which measures the similarity between vocal sample features and eliminates inconsistent ones.

For evaluating the clustering performance of the algorithm, the Fuzzy Partition Coefficient (FPC) and Classification Validity indices are chosen as the metrics. The FPC is defined as follows:

$$NPC=\frac{1}{N}\sum_{i=1}^{N}\sum_{j=1}^{c}\mu_{ij}^{2}$$ (21)

In the text you provided, *N* represents the data points of dataset *K*_*i*_, with a total of C clusters. *U*_*ij*_ denotes the membership degree of the i_th_ data point belonging to the j_th_ cluster. U_ij_ represents the membership degree ranging from 0 to 1, indicating the extent to which a data point is associated with a specific cluster. In the fuzzy c-means algorithm, a data point can be part of multiple clusters, with membership degrees across all clusters equaling 1. The FPC value range is between 0 and 1. When the FPC value approaches 1, it signifies significant differences in the membership degrees of data points among clusters. This suggests that most data points belong to a specific cluster, indicating higher-quality clustering results. Conversely, if the FPC value approaches 1/C, it indicates that the membership degrees of data points among clusters are nearly equal, suggesting less distinct clustering, possibly because the data may not be suitable for being divided into many clusters.

The Classification Validity Index, specifically the Xie-Beni index, is a commonly used fuzzy clustering validity index, especially for FCM clustering. It is defined as:

$$XB=\frac{\sum_{i=1}^{N}\sum_{j=1}^{C}u_{ij}^{2}\times {\left\|x_{i}-v_{j}\right\|}^{2}}{N\times \underset{k\neq l}{\min}{\left\|v_{k}-v_{l}\right\|}^{2}}$$ (22)

where *u*_*ij*_ represents the membership degree of data point *i* to cluster center *j*, *x*_*i*_ represents the data points, *v*_*j*_ represents the cluster centers, *N* is the total number of data points, and *C* is the number of clusters. A smaller XB index indicates tighter clustering within clusters and better separation between clusters, implying better clustering effectiveness.

Clustering method parameters are set as follows: The kmeans++ method is employed to initialize cluster centers, selecting initialization cluster centers that are well separated. In spectral clustering, the similarity matrix is built using the fully connected method with kernel function parameters: gamma is set to 0.01, the degree to 3, and coef0 to 1. For fuzzy c-means clustering, the membership exponent is fixed at 1.2. Both clustering methods are configured to generate 8 clusters, with the maximum number of iterations capped at 1000 and the error threshold established at 0.01. The outcomes of these clustering methods are summarized in the **Table 3**.

**Table 3:** The outcomes of these clustering methods.

Algorithm type	K-means	SC	QAGA-K-means	QPSO-FCM (our methoed)
NPC	0.451	0.472	0.624	0.713
XB	0.51	0.44	0.37	0.25

It can be seen from **Table 3** that the optimized clustering algorithm has obvious advantages in Silhouette Coefficient and CH Score, that is, the same kind of samples are closer than other clustering algorithms, and the different samples are farther apart. Therefore, the QPSO-C-means algorithm proposed in this chapter yields superior performance, leading to a more precise classification model, effectively clustering the eight types of tones- in Yue opera along with their associated phonetic characteristics. Furthermore, the results of optimizing C-means clustering centers using the quantum particle swarm optimization algorithm offer detailed insights into the acoustic features corresponding to the eight-tone categories.

### 3.3 Managing uncertainty interference factors in Yue opera phonetic data

Clustering the phonetic patterns of the eight tonal categories in Yue opera is achievable. Nevertheless, the precision is compromised by the uncertainty interference factor *λ* present at the end of the phonetic data *χ*’. This interference affects the direct application of clustering results in the development of detection algorithms. Therefore, it is essential to eliminate the interference factor A to attain the most accurate phonetic modeling results, which are crucial for the formulation of detection algorithms.

Through in-depth data exploration of the formed clusters, this method initially selects a representative set of samples from each cluster. Subsequently, detailed multidimensional comparisons are performed on the cluster centroid vectors of these samples, utilizing CCF to quantify the correlation between different dimensional features. Through this analysis, we can identify the most highly associated portions within each centroid vector with other vectors, which typically correspond to specific dimensions where the cross-correlation function reaches its peak values. Ultimately, by extracting and retaining the feature vectors on these key dimensions, we obtain a set of concise and effective features. These feature vectors are then utilized for subsequent data model construction to ensure the accuracy and efficiency of the model. This approach not only enhances the interpretability of the model but also optimizes the computational process, making it more adaptable for efficient analysis in complex data environments.

The two sets of data are represented as $x_{i}^{'}=\{x_{i1}^{'},x_{i2}^{'},\cdots ,x_{in}^{'}\}$ and *y* = {*y*_1_,*y*_2_,⋯,*y*_*m*_}, where n ≥ m. The linear correlation operation between $x_{i}^{'}$ and *y* is denoted as $R_{L}(x_{i}^{'},y)$, and its result is also a sequence. The definition of the cross-correlation function is as follows:

$$R(k)=\sum_{j=1}^{m}y_{j}x_{i(j+1)}^{'},k\in [0,1,\ldots ,n-m+1]$$ (23)

In this analysis, we dissect the original vector into a series of sub-vectors, each matching the length of vector y. We then create a cross-correlation series by assessing the similarity between these sub-vectors and y, yielding a series with a total length of n+m-1. Notably, out of this total length, only n-m+1 points are considered informative data points. Among all the R values in the series, the index corresponding to the maximum R-value denotes the starting position of the sub-vector within the original vector that is most similar to y.

**Fig 8:**
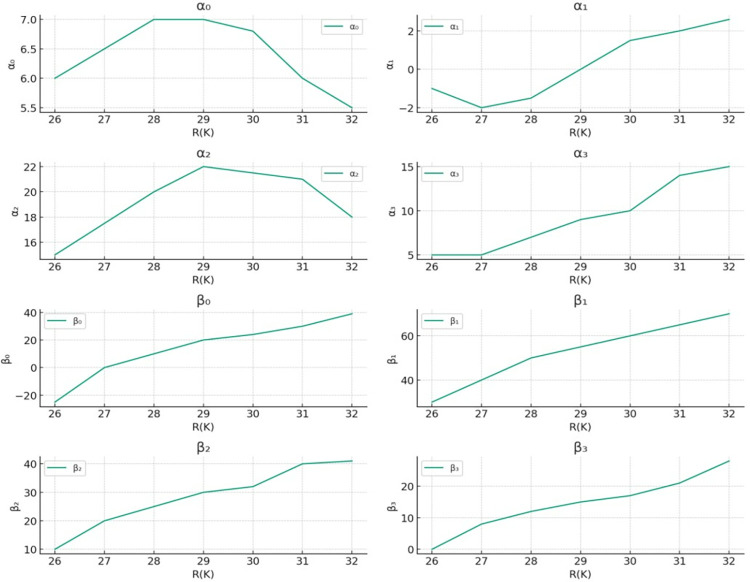
The cross-correlation values corresponding to the different dimensions of the eight-character tune categories in Yue Opera.

The research utilized a cross-correlation function, a variable-length sliding time window, and norm filtering methods to remove unstable elements from cluster centroid feature vectors efficiently. The core strategy of this approach is to retain vital feature information in dimensions where the cross-correlation function reaches its peak while excluding data points that fall outside this threshold. The results depicted in **Fig 8** reveal that, through analyzing cross-correlation values of 8 categories at different k values, only the initial 26 sample features are necessary to eliminate interference factors λ from modeling Yue phonetic tones. By analyzing 36 different clusters, the optimal clustering is found at k = 26, 27,…, 36, which shows a gradual increase in the R(k) value, indicating a sharp increase in clustering effectiveness. Additionally, the first 24 data features are retained for other tone categories as they demonstrate insignificant responses to such interference and thus do not necessitate similar filtering procedures.

**Fig 9:**
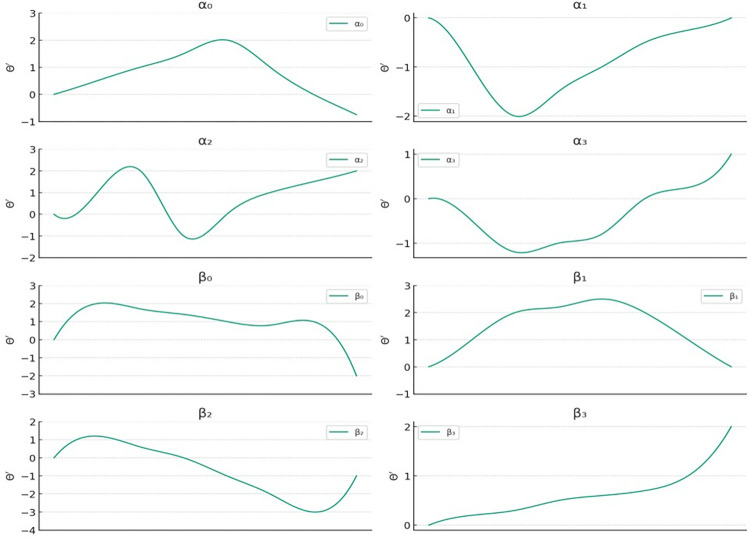
Mitigation of uncertainty disturbance in Yue opera character tone trends.

**Fig 9** illustrates the Yue phonetic tone features obtained using the CCF. By utilizing these experimental findings and their analysis, we have successfully achieved accurate modeling of Yue phonetic tones.

As shown in **Fig 9**, the number of tone schedule changes in each subgraph can be described based on the displayed information. Additionally, **Fig 9** shows the correlation curves between Yue Opera pitch sequences and reference patterns from CCF analysis. Each curve peak represents the highest similarity between the pitch sequence and the reference pattern at specific time lags. This information is vital for understanding the diverse pitch patterns.

α_0_: The number of tone time series begins just below the second interval on the graph, gradually ascending to form a smooth peak at approximately the third interval to the left of the graph’s midpoint. Subsequently, following the peak, the number of tone time series gradually declines, ultimately returning close to its initial value. This trend manifests an overall symmetric shape characterized by a single peak.

α_1_: The number of tone time series initiates slightly below the second interval on the graph, progressively ascending to shape a smooth peak positioned roughly the third interval leftward from the graph’s midpoint. Subsequently, after reaching this peak, the number of tone time series gradually decreases, eventually converging back to its initial value. This trend exhibits an overall symmetric shape distinguished by a single peak.

α_2_: The number of tone time series initiates slightly below the second interval on the graph, progressively ascending to shape a smooth peak positioned roughly the third interval leftward from the graph’s midpoint. Subsequently, after reaching this peak, the number of tone time series gradually decreases, eventually converging back to its initial value. This trend exhibits an overall symmetric shape distinguished by a single peak.

α_3_: The number of tone time series begins slightly above unison, sharply rises to near unison, and then rapidly declines to form a deep trough around the second interval. Subsequently, it ascends again to a positive value at the end, presenting an overall fluctuation resembling an "N" shape.

β_0_: The variation in the number of tone time series in this subgraph remains relatively steady, commencing near 0 degrees. After a minor decline, it rises again, fluctuating throughout the process, not surpassing unison. Ultimately, it returns to a level close to the initial one.

β_1_: The number of tone time series begins in unison, gradually increasing to a peak of approximately a second interval, then declining to nearly 0 degrees. The variation throughout the process is smooth, exhibiting a single arc-shaped trend.

β_2_: The number of tone time series starts slightly above 0 degrees. It descends to the lowest point near the middle of the graph, reaching approximately the third interval, forming a broad U-shaped valley. Subsequently, it ascends to the positive range, demonstrating a distinct "U" shaped variation.

β_3_: This subgraph depicts an almost steadily increasing trend, initiating from 0 degrees and steadily ascending to over a second interval, devoid of prominent peaks or valleys, signifying a gradual upward pattern.

**Fig 7** illustrates the results of applying this clustering method, showing the classification effect of tonal trends among Yue Opera characters. The goal is to enhance the accuracy of Yue Opera vocal style recognition through precise clustering of vocal data. To reduce redundancy-induced interference in tonal transition, the algorithm employs the Cross-Correlation Function (CCF), which measures the similarity between vocal sample features and eliminates inconsistent ones. Figs 8 and 9 display the processed data: **Fig 8** presents cross-correlation values across different dimensions, while **Fig 9** shows the specific tonal trends of Yue Opera characters after processing. Cross-correlation analysis helps in identifying and eliminating unstable factors, thereby improving clustering and tonal trend analysis accuracy. The initial 12 sample features are crucial for eliminating interference as they provide the most consistent information on tonal trends. Dimensions 26 to 32 are excluded due to their susceptibility to instability and noise. Initially, 36 dimensions were considered, but further analysis revealed that only 32 dimensions are useful for final analysis. This dimensional reduction enhances algorithm efficiency and accuracy. The pitch detection algorithm processes the audio data of Yue Opera characters’ voices to detect and analyze tonal patterns, outputting tonal feature values or category labels representing different tonal categories or trends.

### 3.4 Design of pitch feature detection algorithm

**Fig 10:**
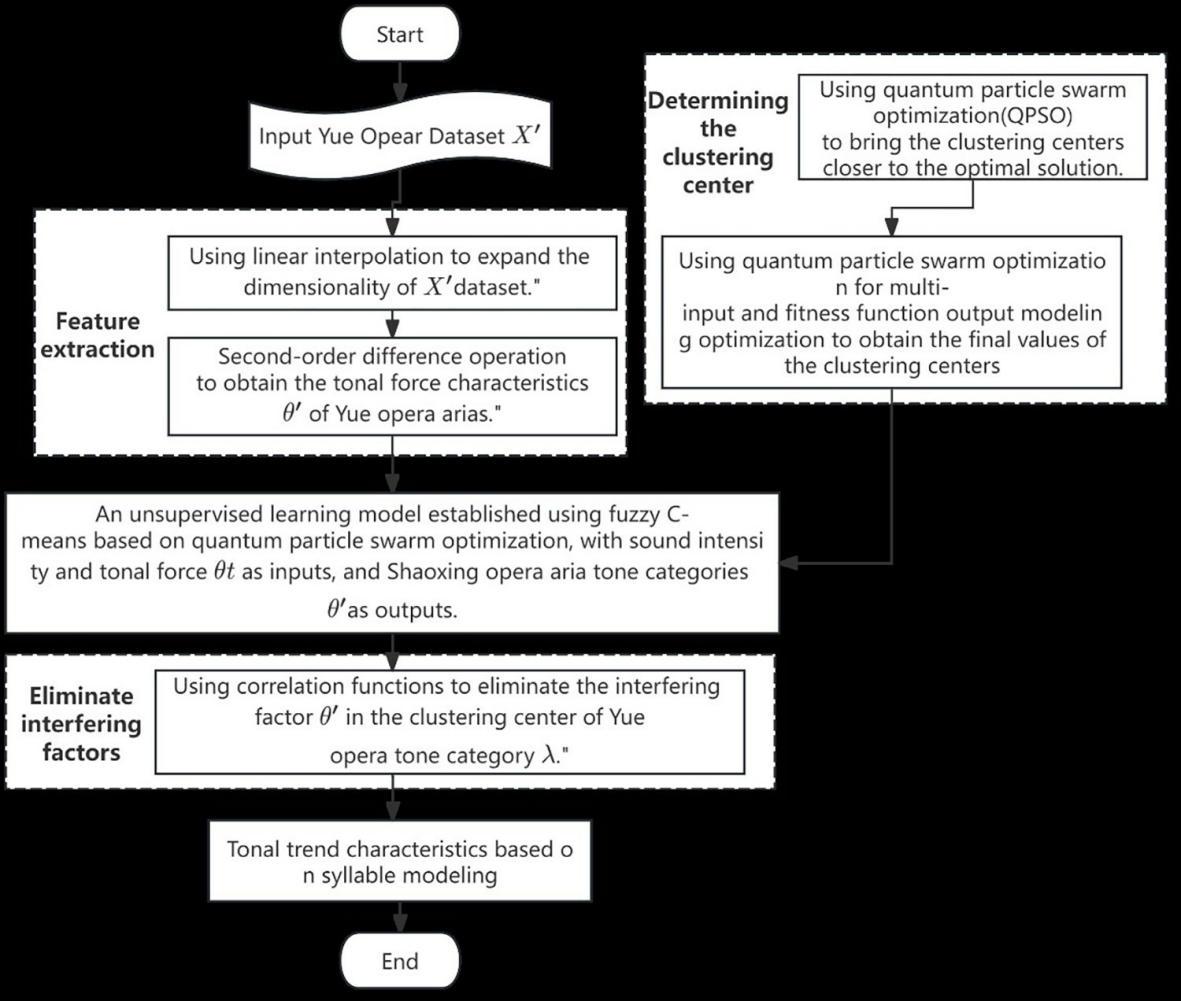
The algorithm flow for modeling the pitch contours of Yue opera phonemes.

Pitch contour data of Yue opera phonemes with standardized dimensions were extracted using feature extraction and optimization methods. Based on this data, the quantum particle swarm algorithm was employed to optimize the clustering centers of fuzzy C-means, followed by clustering using the optimized results. Next, uncertainty interference factors were eliminated using the CCF algorithm, accurately modeling Yue opera phoneme pitch contours. The algorithm flow is depicted in **Fig 10**.

Building upon the precise modeling of Yue opera’s phonetic characteristics outlined above, we apply this understanding to detect whether a piece adheres to Yue opera’s phonetic melody norms.

**Fig 11:**
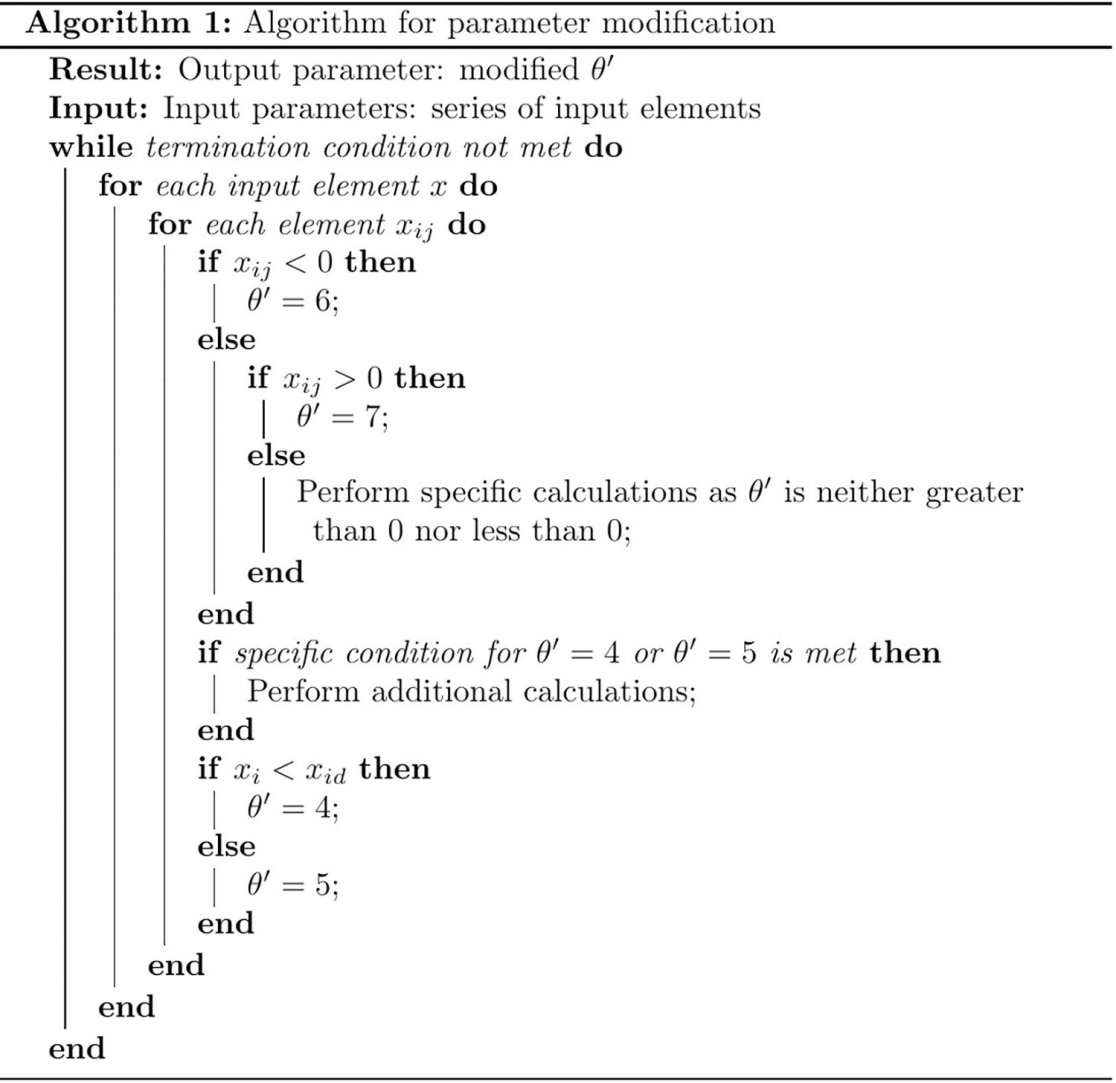
Detection algorithm table for modeling the phonetic characteristics of Yue opera.

The method for this detection is described as follows:

**Fig 11** is designed to demonstrate the application of the aforementioned equation on actual data. The main purpose of this algorithm is to verify the accuracy of our model’s classification of Yueju opera music features through a series of predefined conditions. The input data consists of melodic features extracted in earlier steps, and the output is the cluster centers corresponding to these features. In the formula, $x_{i}^{'}$, represents the i-th sample data in the phonetic melody dataset, and $x_{d_{i}}^{'}$, represents its value on any dimension j (where 0 < j < d’). The detection formula is constructed as follows:

$$f(k)=\arg\min(\sqrt[{p}]{\sum_{j=1}^{n}{\left\|x_{ij}^{'}-u_{kj}^{'}\right\|}^{p}}),k\in [0,1,\cdots ,5]$$ (24)

where $x_{d_{i}}^{'}$ represents the value of the sample data $x_{i}^{'}$ to be detected on the i_th_ feature, $u_{kj}^{'}$ represents the value of the selected cluster center vector of the k_th_ class on the j_th_ feature, where n is the dimensionality (36 in this case), and p is chosen as 2. The primary purpose of Eq (24) is to identify the optimized cluster center closest in Euclidean distance to a test sample, thereby determining the sample’s category. This step follows FCM clustering and QPS optimization to ensure accurate classification of each sample. This section rigorously introduces the theory above, thus completing the foundational framework of the Yue phonetic melody detection algorithm.

## 4. Simulation

This section is dedicated to assessing the efficacy of the Yue character pitch trend detection algorithm, introduced in Chapter 3, through practical experiments using authentic opera score datasets. We aim to ascertain the precision and consistency of the algorithm’s performance. The details of the experimental framework are presented in Section 4.1, while Section 4.2 is devoted to evaluating the precision and reliability of the Yue character pitch category detection algorithm.

### 4.1 The experimental setting

All the Yue singing melodies analyzed in this study originate from authentic Yue opera recordings to analyze and identify different vocal styles and tonal trends in Yue Opera. Key musical segments are extracted from high-quality theater recordings for professional auditory analysis to pinpoint emotionally expressive parts of the singing. These segments are then precisely edited and analyzed for pitch, rhythm, and dynamics, converting these features into numerical data for further study. Yue opera music experts annotate these segments, verifying their artistic and emotional accuracy, thus enhancing our understanding of Yue opera’s musical characteristics. These melodies are transformed into a set of melody data using Eqs (1) and (2), revealing variations in tones for different characters through various pitch combinations. Additionally, these melody data can be mapped onto eight categories of Yue character tones. To more accurately analyze the influence of transitional notes on tone trends, three different Yue works—"Dream of the Red Chamber," "The Butterfly Lovers," and "The Peony Pavilion"—are introduced as sample sources. The study selected eight clusters for analyzing Yue Opera, aligning with traditional categories of character roles defined by voice and performance styles that influence pitch patterns. The cluster selection was guided by cultural and artistic traditions rather than solely by algorithmic criteria. The "Yue Opera Role Pitch Trend Dataset" comprises 2,600 performance pitch sequences, categorized by the characters’ singing styles. This dataset was compiled meticulously after an extensive study of Yue Opera musicology, reflecting the distinct pitch characteristics of various roles and offering insights into the musical complexity of Yue Opera.

### 4.2 Algorithm verification

This section focuses on the classification and detection of Yue character tone trends using Algorithm 1, outlined in Section 3.4, with recognition accuracy as the primary evaluation metric. According to Eq (2), the sample data of character tones are categorized into eight distinct categories: α_0_, α_1_, α_2_, α_3_, β_0_, β_1_, β_2_, β_3_. Through feature optimization and extraction of tone trends, a dataset of character tone trends is acquired and subsequently normalized to range within [–1, 1]. The formula defines the normalization process.

$$\chi_{norm}=\frac{2(\chi^{\prime }-\chi_{min}^{'})}{\chi_{max}^{'}-\chi_{min}^{'}}-1$$ (25)

Here, $\chi_{max}^{'}$ and $\chi_{min}^{'}$ denote the maximum and minimum values in χ′, which, as per **Table 1**, are determined to be 7 and -8, respectively.

**Fig 12:**
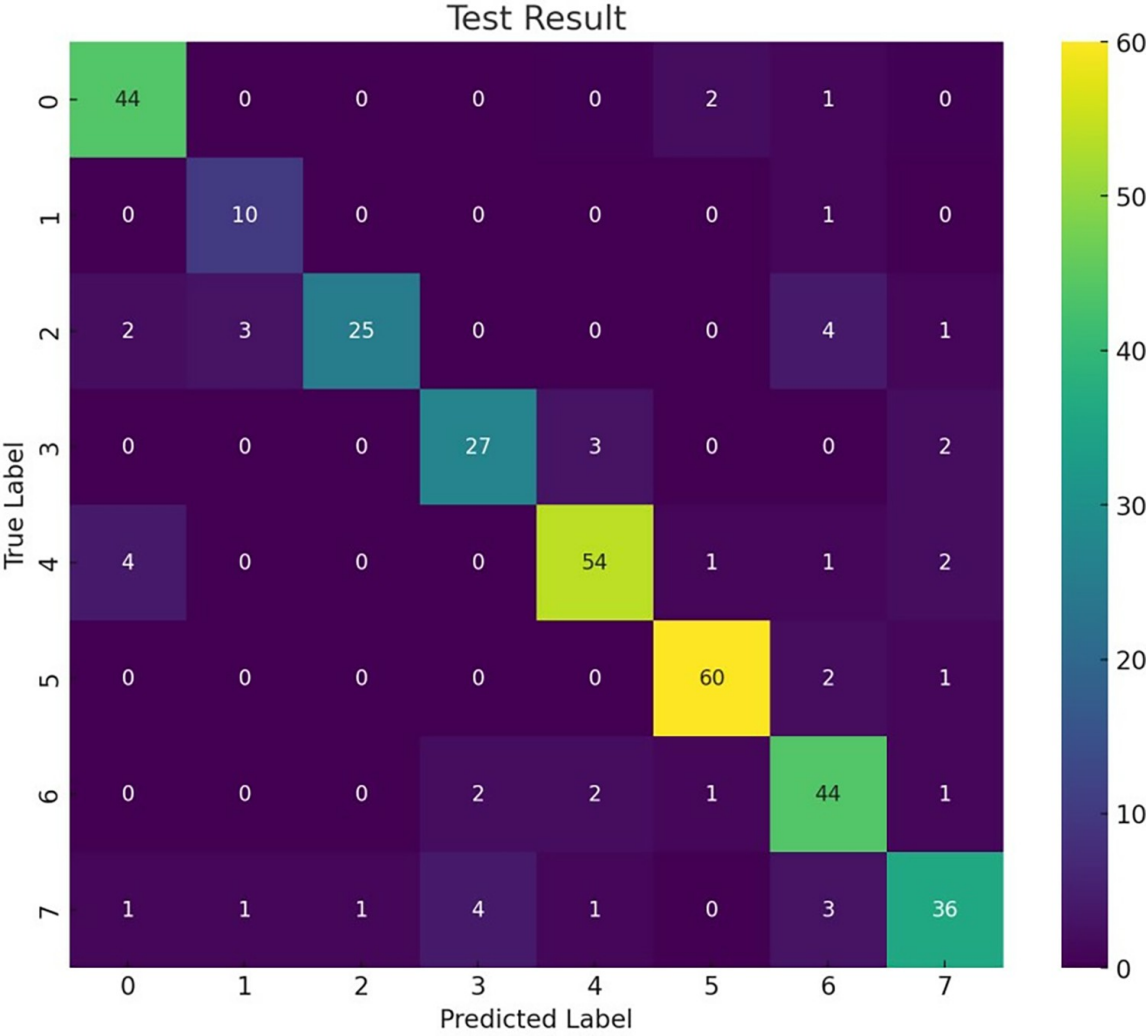
Test result.

This study involved inviting musicology experts to offer detailed annotations for the tone trends *θ*’ in the scores. These experts classified the tone trend data for each character based on their professional knowledge and thorough analysis. Furthermore, 300 new opera score melody samples, distinct from the previous 2600 training samples, were selected for testing. Algorithm 1 was utilized to classify and detect these samples *θ*’, and the resulting classification outcomes are depicted in **Fig 12**. The labels 1 to 8 in the figure correspond to various character tone optimization values in **Table 2**.

Based on the results depicted in **Fig 12**, the detection algorithm proposed in this chapter achieves an overall accuracy of 91.4% when classifying all samples in the test set. Notably, incorrectly identified phonetic melody samples frequently manifest continuous zero values (*θ*_*t*_ = 0) at the beginning and middle positions of the melody. Upon analysis, this phenomenon is attributed to occasional consecutive drag notes with the same pitch distributed at these positions during Yue singing, resulting in a *θ* value of 0 post-feature melody extraction. Moreover, due to the diversity in individual singing styles, Yue drag notes frequently incorporate artistic elements that mirror the characters’ emotions and contexts, such as vocal embellishments, tone quality nuances, dynamics, and ornamentations. These aspects are typically not captured in musical scoring, resulting in feature loss among consecutive zero values (*θ*_*t*_ = 0). Consequently, thoroughly examining this issue is essential, constituting a significant direction for our upcoming research efforts.

## 5. Conclusions

This study introduces a methodology for classifying and identifying Yue opera tone trends. The methodology employs linear and second-order difference operations to map singing tone trends onto the Yue tone melody dataset. Data standardization and tone feature extraction are performed. The study optimizes Fuzzy C-means clustering centers to tackle the challenge of choosing initial clustering centers amid dataset uncertainties. Simulation outcomes indicate that this algorithm achieves results nearer to the optimal solution and exhibits faster convergence than traditional optimization methods. The solution obtained serves as the initial clustering center for fuzzy C-means, with simulations demonstrating the quantum particle swarm algorithm’s superior performance over other clustering methods. Subsequently, the study employs the cross-correlation function algorithm to reduce uncertainty in singing tone trend data, thus enabling accurate Yue tone trend modeling. The study utilizes professionally annotated sample data for algorithm validation as the test set. A limitation of this approach is the necessity for additional research on its effectiveness in identifying artistic expressions like vocal embellishments, tone quality nuances, dynamics, ornamentations, and glissandi in Yue singing. Following the final results, assessments are made to determine if Yue’s works adhere to the rhythmic standards of Yue’s character tones. It should be noted that this study is an initial investigation into the Yue character tone dataset, with future work planned to explore additional issues. In a minor fraction of the Yue character tone trend samples, melodies start or feature mid-sections with two or more consecutive identical pitches. This approach leads to feature loss between consecutive zero values, consequently reducing recognition accuracy. This issue arises from brief embellishments between identical pitches in Yue singing, frequently overlooked in musical compositions. Additional research is planned to tackle this challenge. Moreover, the Yue tone sample data will be enhanced, especially with information on artistic expressions and the styles of different plot characters. Enhancing the dataset can boost the algorithm’s generalizability, improving performance across a broader spectrum of Yue singing contexts. Integrating traditional Yueju music theory knowledge, including scales and tonality, will refine the algorithm and accurately capture Yueju tones’ distinctive features.

## Supporting information

S1 Data (XLSX)

S2 Data (XLSX)

S3 Data (XLSX)

S4 Data (XLSX)

S5 Data (XLSX)

S6 Data (XLSX)

S7 Data (XLSX)

S8 Data (XLSX)
